# Diffusional kurtosis imaging and white matter microstructure modeling in a clinical study of major depressive disorder

**DOI:** 10.1002/nbm.3938

**Published:** 2018-05-30

**Authors:** Kouhei Kamiya, Naohiro Okada, Kingo Sawada, Yusuke Watanabe, Ryusuke Irie, Shouhei Hanaoka, Yuichi Suzuki, Shinsuke Koike, Harushi Mori, Akira Kunimatsu, Masaaki Hori, Shigeki Aoki, Kiyoto Kasai, Osamu Abe

**Affiliations:** ^1^ Department of Radiology The University of Tokyo Tokyo Japan; ^2^ Department of Radiology Juntendo University School of Medicine Tokyo Japan; ^3^ Department of Neuropsychiatry The University of Tokyo Tokyo Japan; ^4^ Department of Radiology The University of Tokyo Hospital Tokyo Japan; ^5^ Department of Radiology IMSUT (The Institute of Medical Science, The University of Tokyo) Hospital Tokyo Japan

**Keywords:** diffusion kurtosis imaging, diffusion tensor imaging, major depressive disorder, microstructure, modeling

## Abstract

Major depressive disorder (MDD) is a globally prevalent psychiatric disorder that results from disruption of multiple neural circuits involved in emotional regulation. Although previous studies using diffusion tensor imaging (DTI) found smaller values of fractional anisotropy (FA) in the white matter, predominantly in the frontal lobe, of patients with MDD, studies using diffusion kurtosis imaging (DKI) are scarce. Here, we used DKI whole‐brain analysis with tract‐based spatial statistics (TBSS) to investigate the brain microstructural abnormalities in MDD. Twenty‐six patients with MDD and 42 age‐ and sex‐matched control subjects were enrolled. To investigate the microstructural pathology underlying the observations in DKI, a compartment model analysis was conducted focusing on the corpus callosum. In TBSS, the patients with MDD showed significantly smaller values of FA in the genu and frontal portion of the body of the corpus callosum. The patients also had smaller values of mean kurtosis (MK) and radial kurtosis (RK), but MK and RK abnormalities were distributed more widely compared with FA, predominantly in the frontal lobe but also in the parietal, occipital, and temporal lobes. Within the callosum, the regions with smaller MK and RK were located more posteriorly than the region with smaller FA. Model analysis suggested significantly smaller values of intra‐neurite signal fraction in the body of the callosum and greater fiber dispersion in the genu, which were compatible with the existing literature of white matter pathology in MDD. Our results show that DKI is capable of demonstrating microstructural alterations in the brains of patients with MDD that cannot be fully depicted by conventional DTI. Though the issues of model validation and parameter estimation still remain, it is suggested that diffusion MRI combined with a biophysical model is a promising approach for investigation of the pathophysiology of MDD.

**Abbreviations used**
AD: axial diffusivity
AK: axial kurtosis
DTI: diffusion tensor imaging
DKI: diffusion kurtosis imaging
FA: fractional anisotropy
GRID‐HAMD‐17: GRID‐Hamilton rating scale for depression, 17‐item version
MD: mean diffusivity
MDD: major depressive disorder
MK: mean kurtosis
NODDI: neurite orientation dispersion and density imaging
ODF: orientation distribution function
RD: radial diffusivity
RK: radial kurtosis
RMSD: root mean squared deviation
SNR: signal‐to‐noise ratio
TBSS: tract‐based spatial statistics

## 1 INTRODUCTION

Major depressive disorder (MDD) is a globally prevalent psychiatric disorder that has a large burden on society.^1^ Although the pathophysiology of MDD has not yet been completely elucidated, its development is believed to be related to a combination of genetic and environmental factors, leading to alterations in the biochemistry, neuroplasticity, and structure of the brain.^2^, ^3^ Many neuroimaging studies have provided evidence that the symptoms of MDD result from disruption of multiple neural circuits involved in emotional regulation.^4^, ^5^, ^6^, ^7^ Diffusion MRI allows us to probe the microstructural properties of tissues at orders of magnitude below nominal image resolution, and this has revealed valuable insights into many neurological and psychological disorders.^8^, ^9^, ^10^ Numerous previous studies using diffusion tensor imaging (DTI) have reported smaller values of fractional anisotropy (FA) in several white matter regions in patients with MDD compared with controls, including the frontal lobe, superior longitudinal fasciculus, anterior limb of the internal capsule, cingulum, and corpus callosum.^10^, ^11^, ^12^

Recently, acquisition with a diffusion weighting higher than in conventional DTI has become clinically feasible. Diffusion kurtosis imaging (DKI) has been proposed as a means of quantifying the deviation from Gaussian diffusion,^13^, ^14^ and it is suitable for the range of diffusion weighting used for typical clinical research (*b*‐value up to 2000‐3000 s/mm^2^). DKI is based on a very general mathematical framework, and therefore it does not rely on strong assumptions about the tissue. It originates from Taylor expansion of the logarithm of the signal in the powers of *b*, and is given as^13^, ^14^

(1) $$ln\frac{S}{S_{0}}=-b\sum_{i,j=1}^{3}g_{i}g_{j}D_{\mathrm{ij}}+\frac{1}{6}{\left(b\bar{D}\right)}^{2}\sum_{i,j,k,l=1}^{3}g_{i}g_{j}g_{k}g_{l}W_{\text{ijkl}}+\ldots$$

where *S*_0_ is the signal at *b* = 0, $\bar{D}$ is the mean diffusivity (MD), and *g*_*i*_ are the components of the unit vector ***g*** in the direction of the applied motion‐probing gradient. *D*_*ij*_ and *W*_*ijkl*_ are the components of the diffusion and kurtosis tensors, respectively. DKI has superior sensitivity for visualizing abnormalities in many brain disorders compared with DTI,^15^, ^16^, ^17^ but studies using DKI to examine MDD are scarce, with only three studies, two in an animal model focusing on gray matter^18^, ^19^ and one in human cerebellum,^20^ being reported.

DTI and DKI are sensitive to many microstructural properties such as neurite density, orientation distribution, and myelination; however, they lack specificity as there is no one‐to‐one relationship to the pathology.^21^ To address this issue, a biophysical model approach was developed in which model parameters, such as axonal volume fraction and compartmental diffusivities, are estimated from the measured diffusion MRI signal. To date, many such models have been proposed^22^, ^23^, ^24^, ^25^, ^26^, ^27^, ^28^ and used in clinical and experimental studies.^29^, ^30^, ^31^, ^32^ However, it is emphasized that so far no definite consensus has been reached on how to parameterize the complex brain microstructure.^33^, ^34^, ^35^ Besides, works by Jelescu et al.,^36^ Novikov et al.,^37^ and Reisert et al.^38^ demonstrated that typical DKI acquisition with a maximum *b*‐value of 2000‐3000 s/mm^2^ is not sufficient to determine all of the parameters, even for a simple two‐compartment model. Novikov et al.^37^ further explained this insufficiency by mathematically showing the need for the *b* ^3^ term in Equation (1). However, extending the series to *b* ^3^ decreases the precision of kurtosis estimation.^39^, ^40^ If our signal is sensitive only up to the *b* ^2^ terms (DKI), additional information or assumption is necessary. Under a specific axially symmetric orientation distribution function (ODF), Jespersen et al.^41^ introduced relationships that link DKI metrics to the model parameters. Though this was originally proposed for the spinal cord and applicability to the brain could be limited, it seems a good candidate option for clinically practical acquisition, as it enables us to take advantage of relatively stable fitting of DKI. Using the same ODF assumption, neurite orientation dispersion and density imaging (NODDI)^27^ has been meaningfully correlated with histology in the brain,^32^, ^42^ though its use of strongly restrictive constraints on the compartmental diffusivities was later called into question.^36^, ^37^, ^43^

Thus, the purpose of the present study was twofold. In the first part of this study, we investigated the microstructural abnormalities in the brains of patients with MDD using DKI combined with tract‐based spatial statistics (TBSS). Then, toward a more specific inference of the underlying microstructural pathology, we conducted a model analysis. Expecting that the axisymmetric ODF would be a good approximation for voxels containing a single fiber population with small dispersion, the corpus callosum was chosen as a target for the model analysis.

## 2 THEORY

### 2.1 Model assumptions

The model used here relies on several widely used assumptions. We list our assumptions below, along with a brief review of supportive and unsupportive studies.

1. The water contributing to the diffusion MRI signal can be divided into two compartments, corresponding to intra‐ and extra‐neurite water. The intra‐neurite compartment consists of mainly axons and, possibly, glial processes. The extra‐neurite compartment encompasses all the other contributions (astrocytes, oligodendroglia, microglia, vasculature, extra‐cellular matrix, etc.), assumed to be in fast exchange. We note that the number of distinguishable compartments is still under debate. For example, a recent study using a cultured cell system^44^ reported that the pre‐exchange lifetimes for both neurons and glial cells were comparable to the diffusion time probed in clinical studies, and questioned the assumption that these cell bodies are in fast exchange with the extra‐cellular space. Though one may prefer to include a third compartment, which is strongly restricted in all directions to represent water trapped inside cell bodies or other small compartments,^22^ recent studies using high *b*‐values^45^ and isotropic diffusion weighting^46^ suggested that the contribution from stationary water would be below the detection limit. Also, though the water within myelin is often neglected in diffusion models because of its short *T*_2_ relaxation time, recent studies^47^, ^48^ have suggested a subtle but significant contribution from the finite exchange with the myelin water pool.
2. The exchange between the two compartments is negligible. The intra‐neurite pre‐exchange lifetime in normal myelinated white matter is reportedly about a second,^49^ and is regarded as sufficiently long compared with the diffusion time.
3. The diffusion time (*t*) is sufficiently long that diffusion within each compartment can be approximated as Gaussian.^50^, ^51^ Recent *in vivo* 52 and *ex vivo* 41 studies observed a diffusion time dependence in the clinically accessible range of *t*. However, this does not necessarily imply that neglecting *q* ^4^ terms is a poor approximation. We may gain more insight into tissue microstructure as well as model validation by studying the functional dependence on *q* and *t* (*b* = *q* ^2^ *t*), though this is beyond the scope of this study.
4. The intra‐neurite compartment can be modeled as a collection of “sticks” (cylinders with zero radius). Neglecting the radius is essentially equivalent to setting the intra‐neurite transverse diffusion coefficient to zero. Recent studies observing asymptotic behavior of the signal at high *b*‐values^45^, ^53^ favored this picture.
5. The extra‐neurite space is approximated by the sum of local anisotropic Gaussian diffusions around each “stick”, described by a diffusion tensor with its principal direction aligned to that of the “stick”.
6. All of the fiber segments within a given voxel have the same values of scalar parameters (compartmental fractions and diffusivities), though these may vary between voxels.
7. The compartmental difference in relaxation time is neglected. While this is for simplification, the accumulation of studies suggested differences in relaxation time between the compartments.^54^, ^55^, ^56^ A recent work^57^ demonstrated that the compartmental fractions are *T*_2_ weighted, and that measurement with variable *T*_E_ is promising for correct model parameter estimation.
  Under these assumptions, the signal is given by^37^, ^38^

(2) $$\begin{array}{l} S\left(b,\hat{g}\right)=\int_{S_{2}}d\hat{n}P\left(\hat{n}\right)K\left(b,\hat{g}\cdot \hat{n}\right),\\ K\left(b,\hat{g}\cdot \hat{n}\right)={\mathrm{fe}}^{-{\mathrm{bD}}_{a}{\left(\hat{g}\cdot \hat{n}\right)}^{2}}+\left(1-f\right)e^{-{\mathrm{bD}}_{e}⊥-b\left(D_{e}∥-D_{e}⊥\right){\left(\hat{g}\cdot \hat{n}\right)}^{2}} \end{array}$$

where *S* denotes the signal normalized to that of *b* = 0, *f* the intra‐neurite signal fraction, and $P\left(\hat{n}\right)$ the fiber ODF. *D*_a_ is the longitudinal intra‐neurite diffusivity. The extra‐neurite diffusivities are *D*_e∥_ and *D*_e⊥_ in the longitudinal and transverse directions, respectively.

### 2.2 Parameter estimation

Toward estimation of parameters in Equation (2), the recently introduced rotationally invariant (“RotInv”) framework^37^, ^38^ gave a way to reduce the dimensionality of the problem by factoring out the ODF. The parameter estimation becomes the following non‐linear least squares problem:^37^

(3) $$\hat{x}=\arg\;\min\;\sum_{l=0,2,\ldots }^{L}\sum_{j=1}^{N_{b}}{\left\{S_{l}\left(b_{j},\hat{x}\right)-p_{l}K_{l}\left(b_{j},\hat{x}\right)\right\}}^{2}$$

with $\hat{x}=\left\{f,D_{a},D_{e∥},D_{e⊥},p_{l}\right\}$ the model parameters to be estimated, and *K*_*l*_ the projections of the kernel $K\left(b,\hat{g}\cdot \hat{n}\right)$ onto Legendre polynomials. The rotational invariants *S*_*l*_ and *p*_*l*_ are defined as

(4) $${S_{l}}^{2}=\sum_{m=-l}^{l}{S_{\mathrm{lm}}}^{2}/4\pi \left(2l+1\right),\;{p_{l}}^{2}=\sum_{m=-l}^{l}{p_{\mathrm{lm}}}^{2}/4\pi \left(2l+1\right)$$

where *S*_*lm*_ and *p*_*lm*_ are spherical harmonic coefficients of the signal and the fiber ODF, respectively. A representative dispersion angle *θ*_disp_ can be computed as $\cos^{2}\theta_{\text{disp}}=\frac{2p_{2}+1}{3}$.^37^, ^38^ For the corpus callosum, histology studies^58^, ^59^ yielded *θ*_disp_ = 14‐22°, corresponding to *p*_2_ ≈ 0.79‐0.91.

For the *b*‐value range in this study, direct fitting of Equation (3) is known to be unstable.^37^, ^57^ Moreover, the problem is bi‐modal; that is, two possible solutions (branches) fit the observed signal equally well.^24^, ^36^, ^37^ Following Novikov et al.,^37^ we label the two solutions +/−. The + branch should be chosen if the ground truth fulfills $4-\sqrt{\frac{40}{3}}<\frac{D_{a}-D_{e∥}}{D_{e⊥}}<4+\sqrt{\frac{40}{3}}$, and the − branch otherwise.^37^

Under the assumption of an axisymmetric ODF, the relationship between the DKI metrics and model parameters was given by Jespersen et al.,^41^

(5) $$\begin{array}{l} 3D_{0}={\mathrm{fD}}_{a}+\left(1-f\right)\left(3D_{e⊥}+\Delta_{e}\right)\\ \frac{3}{2}D_{2}=p_{2}\left\{{\mathrm{fD}}_{a}+\left(1-f\right)\Delta_{e}\right\}\\ {D_{2}}^{2}+5{D_{0}}^{2}\left(1+\frac{W_{0}}{3}\right)={{\mathrm{fD}}_{a}}^{2}+\left(1-f\right)\left(5{D_{e⊥}}^{2}+\frac{10}{3}D_{e⊥}\Delta_{e}+{\Delta_{e}}^{2}\right)\\ \frac{1}{2}D_{2}\left(D_{2}+7D_{0}\right)+\frac{7}{12}W_{2}{D_{0}}^{2}=p_{2}\left\{{{\mathrm{fD}}_{a}}^{2}+\left(1-f\right)\left(\frac{7}{3}D_{e⊥}\Delta_{e}+{\Delta_{e}}^{2}\right)\right\}\\ \frac{9{D_{2}}^{2}}{4}+\frac{35}{24}W_{4}{D_{0}}^{2}=p_{4}\left\{{{\mathrm{fD}}_{a}}^{2}+\left(1-f\right){\Delta_{e}}^{2}\right\} \end{array}$$

where *Δ*_*e*_ ≡ *D*_*e*∥_ − *D*_*e*⊥_, and {*D*_0_, *D*_2_, *W*_0_, *W*_2_, *W*_4_} are obtained from DKI:

(6) $$\begin{array}{l} D_{0}=\frac{1}{3}\left(D_{∥}+2D_{⊥}\right)\\ D_{2}=\frac{2}{3}\left(D_{∥}-D_{⊥}\right)\\ W_{0}=\bar{W}\\ W_{2}=\frac{1}{7}\left(3W_{∥}+5\bar{W}-8W_{⊥}\right)\\ W_{4}=\frac{4}{7}\left(W_{∥}-3\bar{W}+2W_{⊥}\right) \end{array}$$

Equation (5) is essentially equivalent to Equation 18 in Reference 37 up to *b* ^2^. Note that, whereas *D*_∥_ and *D*_⊥_ represent axial diffusivity (AD) and radial diffusivity (RD) in conventional DTI, the kurtosis components, $\left\{W_{∥},W_{⊥},\bar{W}\right\}$, are defined in a different manner from the widely used axial kurtosis (AK), radial kurtosis (RK), and mean kurtosis (MK).^14^ If an axisymmetric ODF is assumed, the conversion between the two definitions can be written^60^

(7) $$\begin{array}{l} \mathrm{AK}=W_{∥}{\bar{D}}^{2}/{D_{∥}}^{2}\\ \mathrm{RK}=W_{⊥}{\bar{D}}^{2}/{D_{⊥}}^{2} \end{array}$$

$\bar{W}$ is the average of the kurtosis tensor over all directions and is defined as^61^

(8) $$\bar{W}\equiv \frac{1}{5}\mathrm{Tr}\left(W\right)$$

Equation (5) is underdetermined since we have six parameters *f*, *D*_*a*_, *D*_*e*∥_, *D*_*e*⊥_, *p*_2_, *p*_4_ but only five independent equations. By further assuming a Watson distribution, the number of parameters is reduced:^41^

(9) $$\begin{array}{l} p_{2}=\frac{1}{4}\left(\frac{3}{\sqrt{\kappa }F\left(\sqrt{\kappa }\right)}-2-\frac{3}{\kappa }\right)\\ p_{4}=\frac{1}{32\kappa^{2}}\left\{105+12\kappa \left(5+\kappa \right)+\frac{5\sqrt{\kappa }\left(2\kappa -21\right)}{F\left(\sqrt{\kappa }\right)}\right\} \end{array}$$

where *κ* is the concentration parameter of the Watson distribution, and *F* is Dawson' function. Because of the Watson ODF assumption, the applicability of this method might be limited to voxels with a single fiber population.

## 3 MATERIALS AND METHODS

### 3.1 Participants

The institutional review board approved this study, and written informed consent was obtained from all participants. Twenty‐six patients with MDD (14 males and 12 females; 39.2 ± 9.9 years old) and 42 age‐ and sex‐matched control subjects (20 males and 22 females; 38.0 ± 8.8 years old) were analyzed. The two groups were also matched for socioeconomic status evaluated using the schooling history of the subjects and their parents. Psychiatric diagnoses were based on the guidelines given in the Structured Clinical Interview for the Diagnostic and Statistical Manual‐IV. The severity of clinical symptoms was measured with the GRID‐Hamilton rating scale for depression, 17‐item version (GRID‐HAMD‐17).^62^ The demographic and clinical characteristics of the study subjects are provided in Table 1. Patients with a history of neuropsychological disease other than MDD, alcohol or drug abuse, head trauma with accompanying loss of consciousness, or signal abnormalities on conventional diagnostic MRI were not included. All control subjects were screened to exclude any psychiatric disorders using the Japanese version of the Mini International Neuropsychiatric Interview^63^ by trained psychiatrists.

**Table 1:** Demographic and clinical characteristics of the study subjects (mean ± standard deviation)

	Patients with MDD	Healthy volunteers
Male/female	14/12	20/22
Age (years)	39.2 ± 9.9	38.0 ± 8.8
Education (years)	15.3 ± 2.2	15.9 ± 2.4
Age of onset (years)*	28.3 ± 11.8	
Disease duration (years)*	9.6 ± 7.2	
Imipramine‐equivalent dose (mg)	130.9 ± 150.6	
GRID‐HAMD‐17	12.1 ± 6.1	

* Time of onset could not be specified in three patients.

### 3.2 Image acquisition and processing

Diffusion MRI data was acquired using a 3 T unit (Discovery MR750w; GE Healthcare, Milwaukee, WI, USA). A single‐shot, spin‐echo, echo planar imaging sequence was used with three diffusion weightings (*b* = 1000, 1500, and 2000 s/mm^2^) along 30 non‐collinear directions, and five *b* = 0 s/mm^2^ volumes (*T*_R_ = 13 000 ms; *T*_E_ = 86.1 ms; voxel size = 1.88 × 1.88 × 2.50 mm^3^). Diffusion gradient length (*δ*) and spacing (*Δ*) were held constant (*δ*/*Δ* = 35.1/44.7 ms). Raw images were denoised,^64^ corrected for Gibbs ringing,^65^ and corrected for eddy currents and motions using the *eddy* tool in the FMRIB Software Library (FSL Version 5.0.9).^66^ Smoothing was not applied. DTI and DKI parameters (AD, RD, MD, FA, AK, RK, and MK) were calculated using the weighted linear least squares estimation (https://github.com/NYU-DiffusionMRI/Diffusion-Kurtosis-Imaging).^67^ We used constraints: *D*_app_ > 0, 0 < *K*_app_ < *b*_max_/(3*D*_app_),^68^ where *D*_app_ and *K*_app_ are the directional apparent diffusion coefficient and apparent kurtosis, respectively.

### 3.3 TBSS analysis

Voxel‐wise statistical analysis was carried out using the TBSS tool in FSL. All subjects’ FA images were aligned into a common space by means of nonlinear registration, followed by creation of a mean FA skeleton. The other diffusion metric maps (AD, RD, MD, AK, RK, and MK) were also projected onto the mean FA skeleton by applying the same transformation. Voxel‐wise statistics of the skeletonized data were analyzed with the *randomise* tool in FSL to test for group differences, with age and sex as nuisance covariates. We used 10 000 permutations, and statistical inference was conducted using threshold‐free cluster enhancement with statistical significance set at *p* < 0.05, corrected for multiple comparisons.

### 3.4 Model analysis

#### 3.4.1 Model parameter estimation

We calculated model parameters from the DTI and DKI metrics under the assumption of a Watson ODF (Method 1). After substituting Equation (9) into Equation (5), we solved the first four equations to express *f* and all diffusivities in terms of *p*_2_(*κ*), as instructed in Reference 37. The aforementioned two branches emerge in this process. Then, we determined *κ* by solving the fifth equation numerically within the interval 0 ≤ *p*_2_ ≤ 1, and obtained two sets of parameters $\left\{f,D_{a},D_{e∥},D_{e⊥},\kappa \right\}\pm$. Outcomes outside a plausible range (0 ≤ *f*, *p*_2_ ≤ 1, and 0 ≤ *D* ≤ 4 for all diffusivities) were removed for the subsequent analyses. The upper bound of diffusivities was relaxed to 4 μm^2^/ms instead of 3 μm^2^/ms (free water diffusivity at the body temperature) to account for possible overshoot due to noise, artefacts, and bias in estimation.

We also performed the straightforward nonlinear fitting of Equation (3) up to *L* = 2,^37^, ^38^ Method 2, as a reference method not biased by the ODF assumption and arbitrary branch choice. For each voxel, fitting was initiated from a randomly chosen starting point (within 0 ≤ *f*, *p*_2_ ≤ 1, 0 ≤ *D* ≤ 3). A box constraint was imposed: 0 ≤ *f*, *p*_2_ ≤ 1, 0 ≤ *D* ≤ 4. The spherical harmonic coefficients of the signal for each *b*‐value shell were computed using the MRTrix3 package (J‐D Tournier, Brain Research Institute, Melbourne, Australia, https://github.com/MRtrix3/mrtrix3).^69^ The model fitting was performed with MATLAB (MathWorks, Natick, MA, USA).

#### 3.4.2 Numerical simulation

Numerical simulation was performed to evaluate quality of parameter estimation. Virtual diffusion MRI signals were generated using Equation (2). Each “voxel” contained 30 identical fiber segments with randomly assigned orientations without imposing axisymmetry. The acquisition protocol matched that of the human data. Rician noise was added so that the signal‐to‐noise ratio (SNR) for *b* = 0 s/mm^2^ was 25. Each simulation was performed with 10 000 random combinations of ground truth values distributed uniformly within biophysically relevant intervals (0.1 ≤ *f* ≤ 1, *p*_2,min_ ≤ *p*_2_ ≤ 1, 0.5 ≤ *D*_a_, *D*_e∥_ ≤ 3, 0.1 ≤ *D*_e⊥_ ≤ 1.5), where *p*_2,min_ was varied from 0 to 0.9 with a step of 0.1. For each virtual voxel, parameter optimization was carried out in the same manner as in the human data. For Method 1, the branch choice was based on the ground truth values. We evaluated estimation error using the root mean squared deviation (RMSD) from the ground truth.

Next, to see the issues of accuracy and precision separately, the simulation was run with 1000 different noise realizations using a fixed ground truth. For underlying values, we chose *f* = 0.7, *D*_a_ = 2.2 μm^2^/ms, *D*_e∥_ = 1.8 μm^2^/ms, *D*_e⊥_ = 0.5 μm^2^/ms, and *p*_2_ = 0.7, in agreement with the values reported using richer acquisition including higher *b*‐values,^37^, ^45^ variable *T*_E_ values,^57^ and multiple diffusion encoding.^46^, ^70^ We report the accuracy and precision using

$$\begin{array}{l} \text{relative}\;\text{mean}\;\text{signed}\;\text{difference}=\frac{〈\text{estimation}〉‐\text{ground}\;\text{truth}}{\text{ground}\;\text{truth}}\\ \text{relative}\;\text{standard}\;\text{devation}=\frac{\mathrm{std}\;\left(\text{estimation}\right)}{\text{ground}\;\text{truth}} \end{array}$$

respectively, where <⋅> refers to the average over the noise realizations.

#### 3.4.3 Comparison between the groups

**Figure 1:**
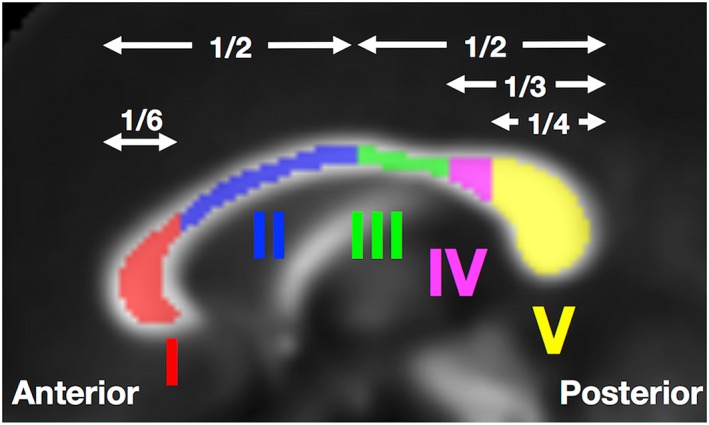
Regions of interest in the corpus callosum were defined according to a previously proposed scheme.^71^ The callosum was segmented into five parts: Region I, prefrontal; Region II, premotor and supplementary motor; Region III, motor; Region IV, sensory; Region V, parietal, temporal, and occipital

Regions of interest were defined in the midsagittal plane of the mean FA image obtained from the TBSS process, and then warped into the native subjects’ space. The corpus callosum was segmented into five parts according to the scheme by Hofer and Frahm^71^ (Figure 1): Region I, prefrontal; Region II, premotor and supplementary motor; Region III, motor; Region IV, sensory; Region V, parietal, temporal, and occipital. To mitigate partial volume effects with CSF, a threshold of FA ≥ 0.5 was imposed. Model parameters extracted from the five regions were compared between the patients with MDD and the controls using a two‐sided *t*‐test, assuming unequal variances. Correlations with GRID‐HAMD‐17, disease duration, and antidepressant medication dose were examined using Spearman' rank correlation coefficient. The statistical significance threshold was defined as *p* = 0.05. As Method 2 is not dependent on specific ODF shapes and therefore generalizable to the whole brain, its outcomes were also analyzed with TBSS.

## 4 RESULTS

### 4.1 DKI‐TBSS

**Figure 2:**
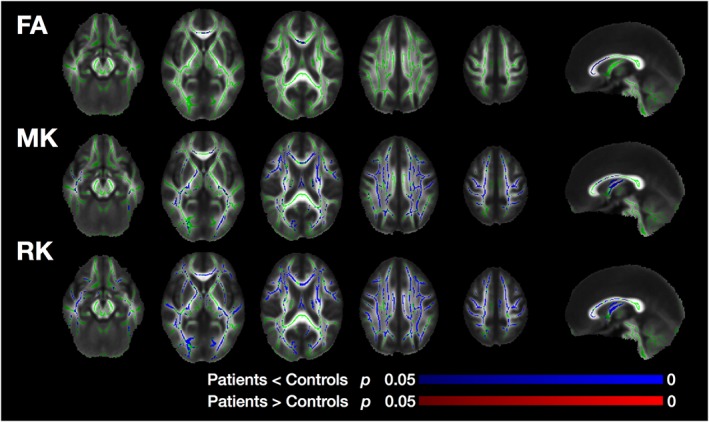
Results of TBSS comparing the brains of patients with MDD and controls. Mean FA skeleton (green) overlaid on the mean FA map. Voxels with significantly greater/smaller values in the patients are shown in red/blue. The significance threshold was defined as *p* < 0.05, corrected for multiple comparisons

The patients with MDD showed significantly smaller FA values in the genu and frontal portion of the body of the callosum, which is consistent with previous studies (Figure 2).^10^, ^11^, ^12^ The patients also had smaller values of MK and RK, but, compared with FA, MK and RK abnormalities were distributed more widely, predominantly in the frontal lobe but also in the parietal, occipital, and temporal lobes. Within the callosum, the regions with smaller MK and RK were located more posteriorly than the region with smaller FA. No significant differences in AD, RD, MD, or AK were observed.

### 4.2 Model analysis

**Figure 3:**
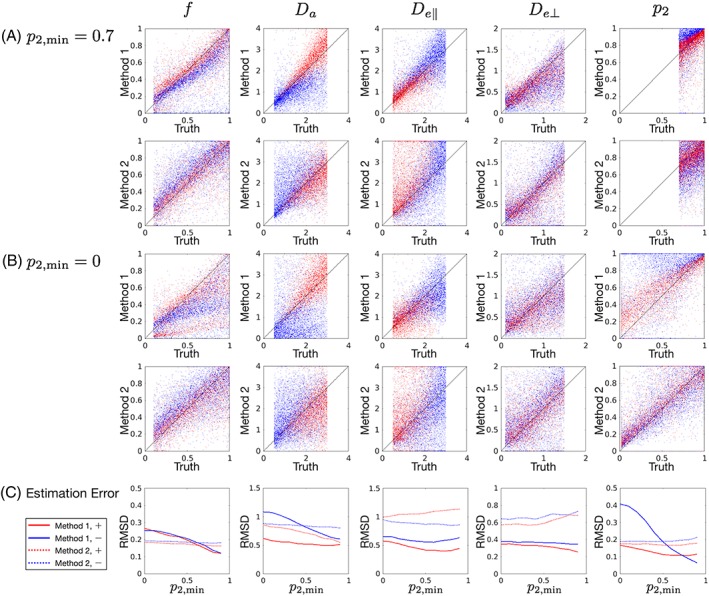
A,B, Scatter plots demonstrating correlations between the ground truth and the estimated values in the simulation. C, Estimation error evaluated by RMSD. Red/blue colors correspond to +/− branches assigned based on the ground truth values. Method 1 had smaller RMSD than Method 2 for a wide range of ground truth parameters, but its advantage decreased with the increase of underlying dispersion (decrease of *p*_2,min_)

**Figure 4:**
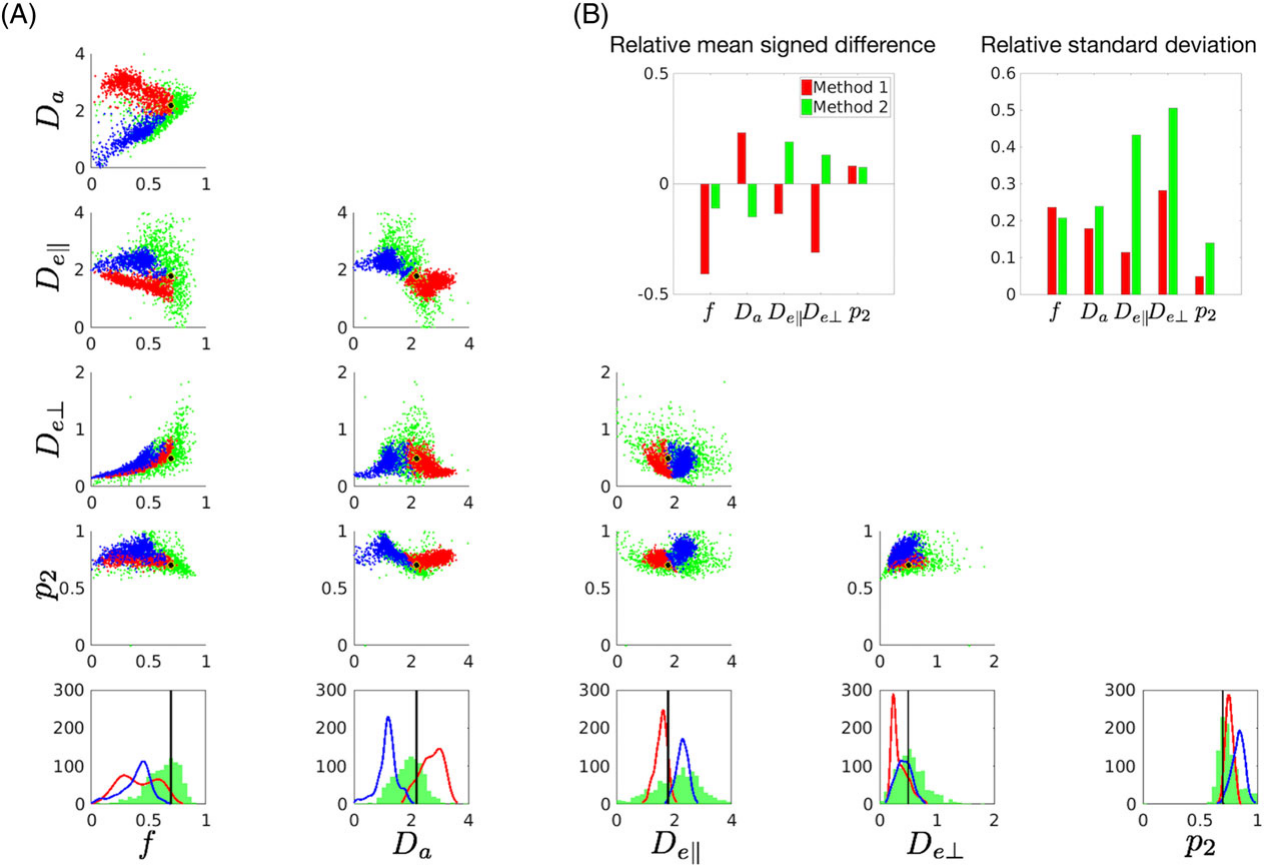
A, Scatter plots between all estimated parameters in the simulation with fixed ground truth. Red/blue colors correspond to +/− solutions in Method 1, and green corresponds to Method 2. The histograms at the bottom show the distributions of the outcome values. The black dots/lines indicate the ground truth. Though the ground truth chosen here corresponds to the + branch, solutions using both branches are shown for Method 1. Spurious correlations are observed between parameters, in different ways depending on the estimation techniques. B, Accuracy and precision are shown for each model parameter estimated with Method 1 and Method 2

Figure 3 illustrates the parameter estimation error. The quality of estimation was dependent on *p*_2,min_, and on the branch to which the ground truth corresponded. It was suggested that *f* and *p*_2_ could be obtained reasonably well from the present acquisition for the range of dispersion expected in the corpus callosum (*p*_2_ ≈ 0.79‐0.91),^58^, ^59^ but estimation of compartmental diffusivities was poor. Though Method 1 had smaller RMSD than Method 2 for a wide range of underlying parameters, its advantage decreased with the increase of dispersion (decrease of *p*_2,min_). Figure 4 demonstrates the accuracy and precision for the specific ground truth setting. Overall, though Method 1 had strength in precision, greater estimation bias was observed, probably because of the ODF assumption and error in kurtosis tensor estimation.^39^, ^40^ What we saw for Method 2 had been already investigated in more detail by the prior studies,^37^, ^38^, ^57^ but is shown here for the particular acquisition in this study. Supplementary Figure 1 shows the same as Figure 3B, but colored with FA and MK. It seems difficult to set some sort of appropriateness criteria for Method 1 based on FA or MK, when we do not have a good knowledge of the ground truth, such as for *p*_2_ in the corpus callosum.

**Figure 5:**
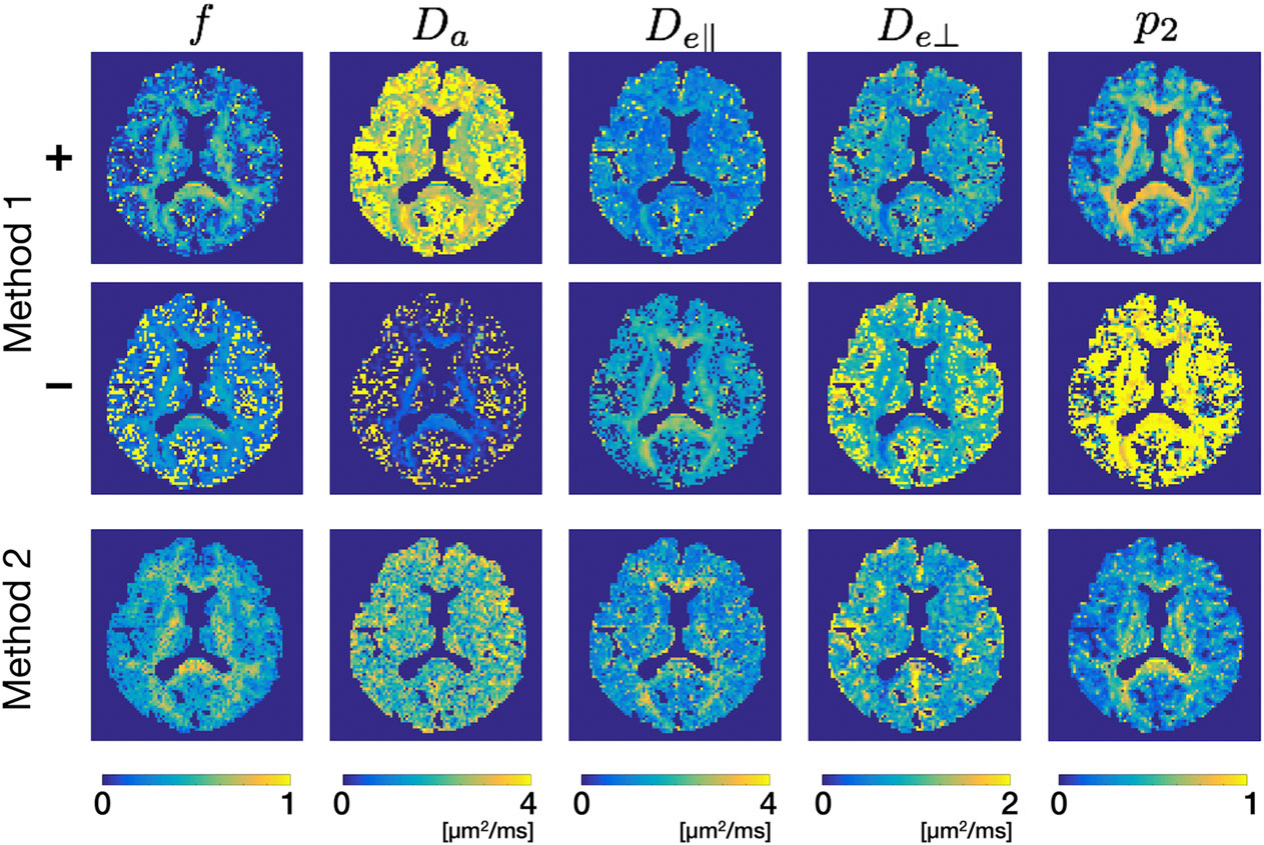
Parameter maps in a representative subject. While the contrast looks reasonable with visual inspection except for the exceedingly large *p*_2_ in the − branch with Method 1, the differences between the methods observed even in the corpus callosum and the low precision indicate the limitation of the present model analysis

**Figure 6:**
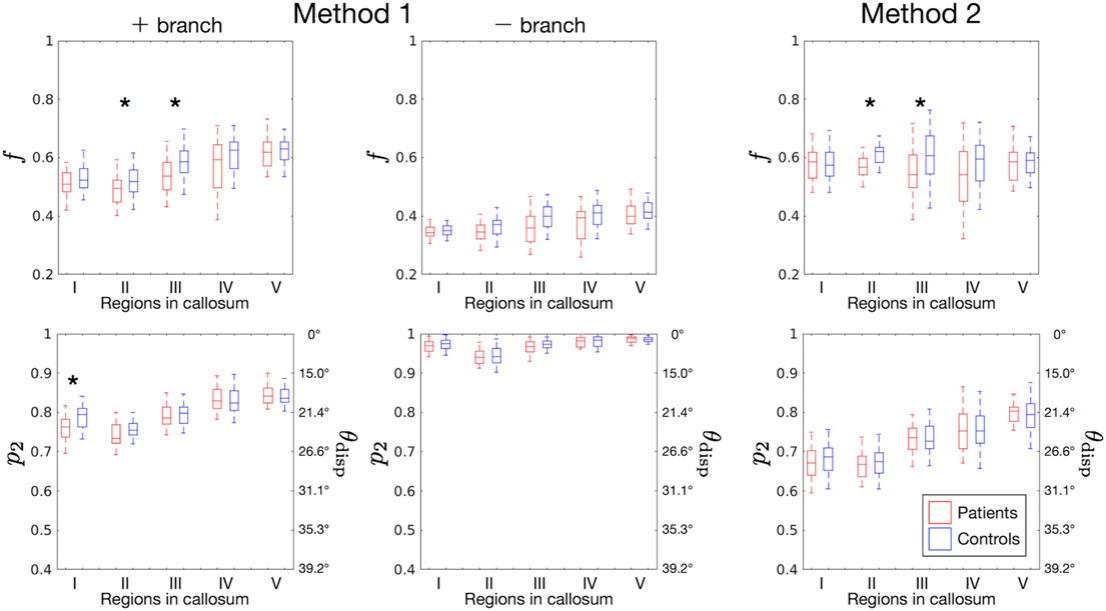
Box‐and‐whisker plots of intra‐neurite signal fraction (*f*) and representative fiber dispersion angle (*θ*_disp_, computed from *p*_2_). Based on previous histology studies reporting *θ*_disp_ = 14‐22° and *f* = 0.6‐0.8,^42^, ^58^, ^59^, ^72^ the + branch was chosen for Method 1. *Statistically significant difference (*p* < 0.05, two‐sided *t*‐test with unequal variances)

**Figure 7:**
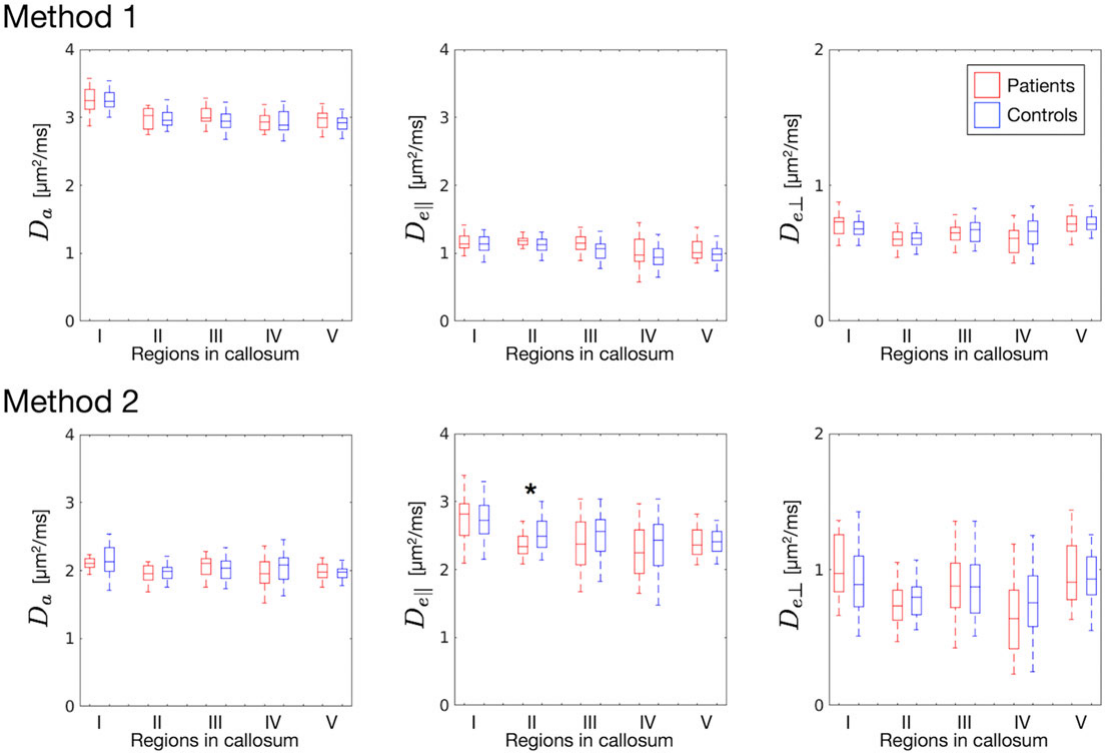
Box‐and‐whisker plots of diffusivity measures (*D*_a_, longitudinal intra‐neurite diffusivity; *D*_e∥_ and *D*_e⊥_, extra‐neurite diffusivities in the parallel and transverse directions, respectively). *Statistically significant difference (*p* < 0.05, two‐sided *t*‐test with unequal variances)

Figure 5 shows parameter maps in a representative subject. Box‐and‐whisker plots of *f* and *p*_2_ for all subjects (Figure 6) showed that, for Method 1, the values in the + branch were more similar to those reported from histology (*θ*_disp_ = 14‐22° and *f* = 0.6‐0.8)^42^, ^58^, ^59^, ^72^ than those in the − branch. We therefore used this branch for Method 1. Both methods demonstrated significantly smaller values of *f* in Regions II and III (*p* < 0.05) in the patients with MDD compared with controls. Method 1 showed significantly greater orientation dispersion in the patients in Region I (*p* < 0.001). Significantly smaller *D*_*e*∥_ (*p* = 0.02) was seen in Region II with Method 2, but this was not supported with Method 1 (Figure 7). We did not find any significant correlations with GRID‐HAMD‐17, disease duration, or antidepressant medication dose.

**Figure 8:**
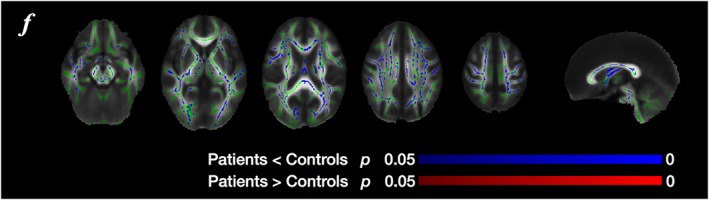
Results of TBSS using the outcomes of Method 2. Voxels with significantly smaller values of *f* had spatial distributions similar to those of MK and RK in Figure 2. The significance threshold was defined as *p* < 0.05, corrected for multiple comparisons

Figure 8 shows results of TBSS using the outcomes of Method 2. Voxels with significantly smaller values of *f* in the patients had spatial distributions similar to those of MK and RK. We did not observe any significant differences in the other parameters.

Supplementary Figure 2 demonstrates correlations among the model parameters in Region II. The trend was similar in the other regions of interest. The correlations closely resembled those induced by noise (Figure 4), suggesting that they are not particularly related to the biological reality, but intrinsic to the estimation methods.

## 5 DISCUSSION

Here we found that the brains of patients with MDD had smaller values of MK and RK compared with controls. The differences were predominantly in the frontal lobe but were also in the parietal, occipital, and temporal lobes, showing more extensive distribution than that of FA. Aiming to achieve a more specific interpretation of these observations, we conducted a biophysical model analysis. Smaller values of intra‐neurite signal fraction were observed in the body of the callosum. Also, we found greater orientation dispersion in the genu of the callosum. Our findings suggest that DKI can provide information that complements conventional DTI with regard to the pathophysiology of MDD, both in the spatial distribution of the abnormalities and with reference to the background pathology.

The extensive DKI abnormalities observed in the present study are consistent with the concepts of MDD as a network disorder caused by disruption of multiple neuronal circuits. Cumulative evidence from network analyses, both functional and structural networks, suggests that MDD manifests as an impairment of the default mode network, frontostriatal‐limbic circuit, executive network, and salience network.^4^, ^6^, ^7^ In addition, MRI volumetric studies have demonstrated widespread gray matter atrophy in the ventrolateral and ventromedial frontal systems, parahippocampal and fusiform gyri, hippocampus, thalamus, and some areas of the parietal lobes and cerebellum.^73^ Compared with the findings in these modalities, the abnormalities reported from DTI voxel‐wise analyses seem disproportionally small in distribution.^74^ The present results suggest that DKI is capable of demonstrating abnormalities that are not fully depicted in DTI.

Considering the remaining ambiguity in model validity and large estimation error, the present results from model analysis should not be taken too literally. Nevertheless, our observation of smaller intra‐neurite signal fraction in the patients was compatible with histology studies in human^75^ and animal models^76^, ^77^ that highlighted changes in the prefrontal white matter in terms of decreased oligodendrocyte density and function, reduction of myelin, and structural abnormalities of nodes of Ranvier. These changes affect axonal physiological activities,^76^ and lead to disruption of axon maintenance and eventually axonal atrophy.^77^ The electron‐microscopic findings in a mouse model include excessive arborization of oligodendrocytic processes and narrowing of nodes of Ranvier.^76^ Excessive arborization is expected to result in greater dispersion measured by diffusion MRI, which is again compatible with our findings. Indeed, Region I matches the location where the stress‐induced abnormalities have been reported in mice,^76^ and connects brain regions in the two hemispheres that are important for emotional and cognitive functions.

However, the particularly poor accuracy and precision of the compartmental diffusivities restricts our interpretations. Excessive arborization and narrowing of nodes of Ranvier are expected to lead to greater hindrance of the extra‐neurite diffusion. Our results are not very convincing in this perspective. Though smaller values of *D*_e∥_ were seen with Method 2 in Region II, the large estimation error warns against relating this to underlying pathology. The accuracy of diffusivities in this study is also questionable in view of recent evidence that suggested *D*_a_ ≥ *D*_e∥_ and *D*_a_ ≈ 1.9‐2.2 μm^2^/ms.^34^, ^35^, ^37^, ^45^, ^46^, ^57^, ^70^ Though *D*_a_ by Method 2 was close to the expected range, the observed *D*_a_ ≤ *D*_e∥_ trend possibly indicates that our estimation might have fallen into the wrong branch or some spurious local minima^36^, ^37^ due to the low maximum *b*‐value and SNR. Thus, for now, it is not clear whether the absence of significant differences in either *D*_e∥_ or *D*_e⊥_ was a sign of inappropriate model choice or solely due to our limited sensitivity to these parameters. The issues of model design and parameter estimation are currently under debate, and we have to wait for further model validation and development of optimal acquisition designed for the verified model. Promising results have been already reported using extended acquisitions including higher *b*‐values,^37^, ^45^ variable *T*_E_ values,^57^ or multiple diffusion encoding,^43^, ^46^, ^70^ while the heavy demand on hardware and acquisition time is a problem for application in clinical research.

The present study has several limitations. First, the effect of medication is a possible confounding factor, a majority of the patients were already medicated at the time of the MRI. Second, we could not provide evidence that the employed model assumptions actually held in our subjects. Although we took care to select a model that is not overly simplistic, the outcome could be biased if the assumptions were violated. For example, if the patients with MDD are suffering from disruption of oligodendrocytes and myelin,^75^, ^76^, ^77^ inter‐compartment exchange may be non‐negligible, and may have led to under‐estimation of intra‐neurite fraction in the patients.^78^ Also, we emphasize that the model parameters in this study are considered as dependent on acquisition as described in Section 2.1, and therefore not very specific to the underlying tissue properties in a strict sense. For example, because the compartment signal fractions were *T*_2_ weighted,^57^ the observed differences in *f* may reflect differences in the *T*_2_ values, not in the actual volume fractions. For Method 1, the branch choice was made based on evidence from healthy brains, but the correct branch might be different among subjects as well as among voxels. Third, we lack comparison with pathology or neurophysiological tests in the studied population to support our interpretation of diffusion MRI findings.

In conclusion, this study has shown that DKI is capable of demonstrating microstructural alterations in the brain of patients with MDD that cannot be fully depicted by conventional DTI. The model analysis suggested smaller intra‐neurite signal fraction and greater orientation dispersion in the corpus callosum of the patients, which was consistent with the existing literature of white matter pathology in MDD. We also reported that our ability to infer specific microstructural properties from the present acquisition was limited. Taken together, our results suggest that diffusion MRI combined with the biophysical model is a promising approach for investigation of the pathophysiology of MDD, and future studies incorporating fruit from the ongoing progress in diffusion microstructural imaging are warranted.

## DISCLOSURE STATEMENT

The authors have no actual or potential conflicts of interest.

## Supporting information


**Fig. S1** Scatter plots showing the same results as Figure 3b, but colored with **a.** FA and **b.** MK, respectively. It seems difficult to set useful quantitative criteria for applicability of Method 1 based on FA or MK, as such threshold would be very stringent, like FA > 0.8.
**Fig. S2** Correlations among the model parameters in Region II of the corpus callosum. The trend was similar in the other ROIs. The correlations were dependent on the methods used and closely resembled those induced by noise (Figure 4), suggesting these correlations are intrinsic to our parameter estimation and not much related to the biological reality.
