# Supplementary material for: Diffusional kurtosis imaging and white matter microstructure modeling in a clinical study of major depressive disorder
Source: NMR Biomed. 2018 May 30;31(7):e3938. doi: 10.1002/nbm.3938 (PMC6032871; doi:10.1002/nbm.3938)
Supplement: Supplementary file 1 — Fig. S1 Scatter plots showing the same results as Figure 3b, but colored with a. FA and b. MK, respectively. It seems difficult to set useful quantitative criteria for applicability of Method 1 based on FA or MK, as such threshold would be very stringent, like FA > 0.8. Fig. S2 Correlations among the model parameters in Region II of the corpus callosum. The trend was similar in the other ROIs. The correlations were dependent on the methods used and closely resembled those induced by noise (Figure 4), suggesting these correlations are intrinsic to our parameter estimation and not much related to the biological reality. [file NBM-31-na-s001.docx]

**
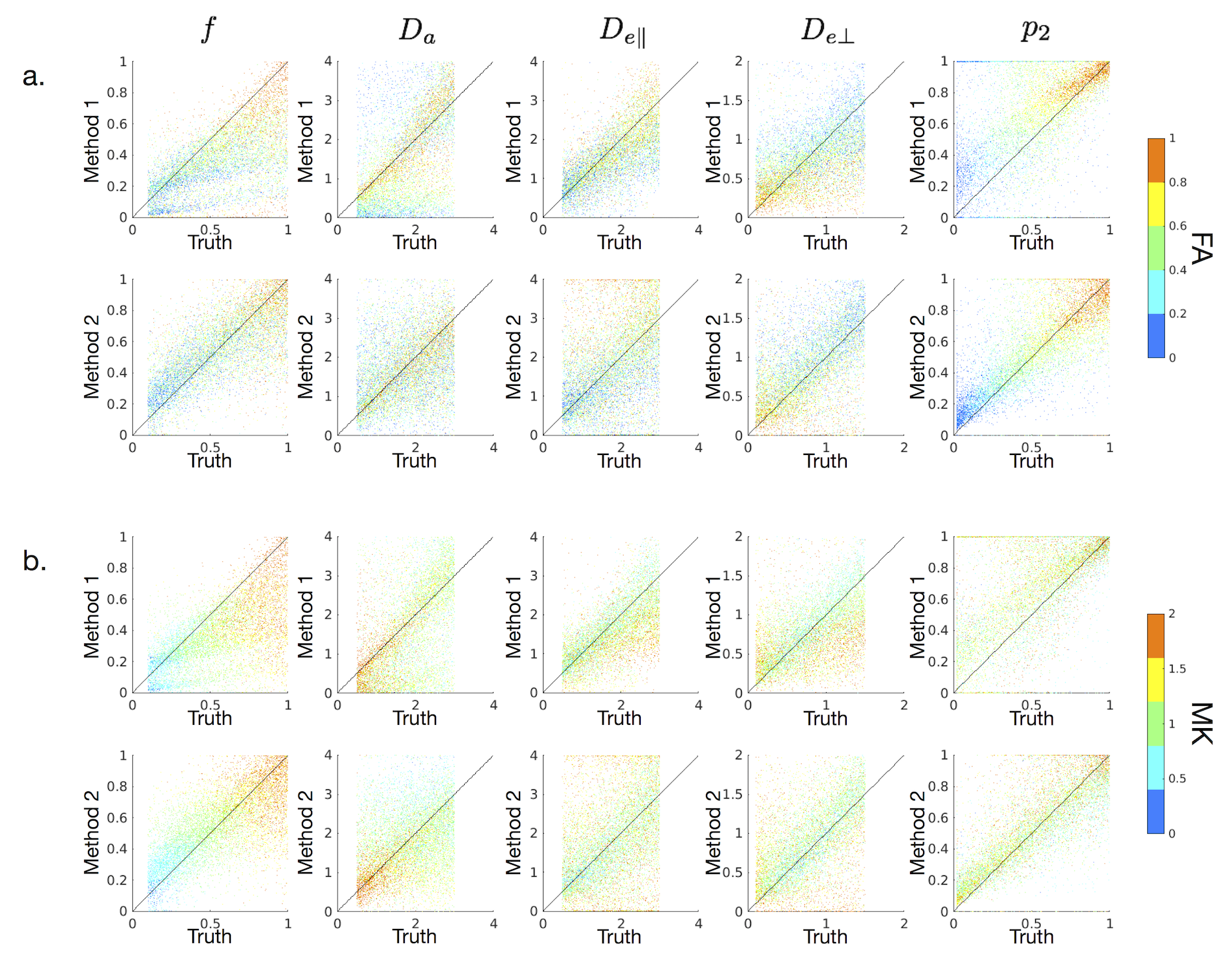
**

**Fig. S1** Scatter plots showing the same results as Figure 3b, but colored with **a.** FA and **b.** MK, respectively. It seems difficult to set useful quantitative criteria for applicability of Method 1 based on FA or MK, as such threshold would be very stringent, like FA > 0.8.


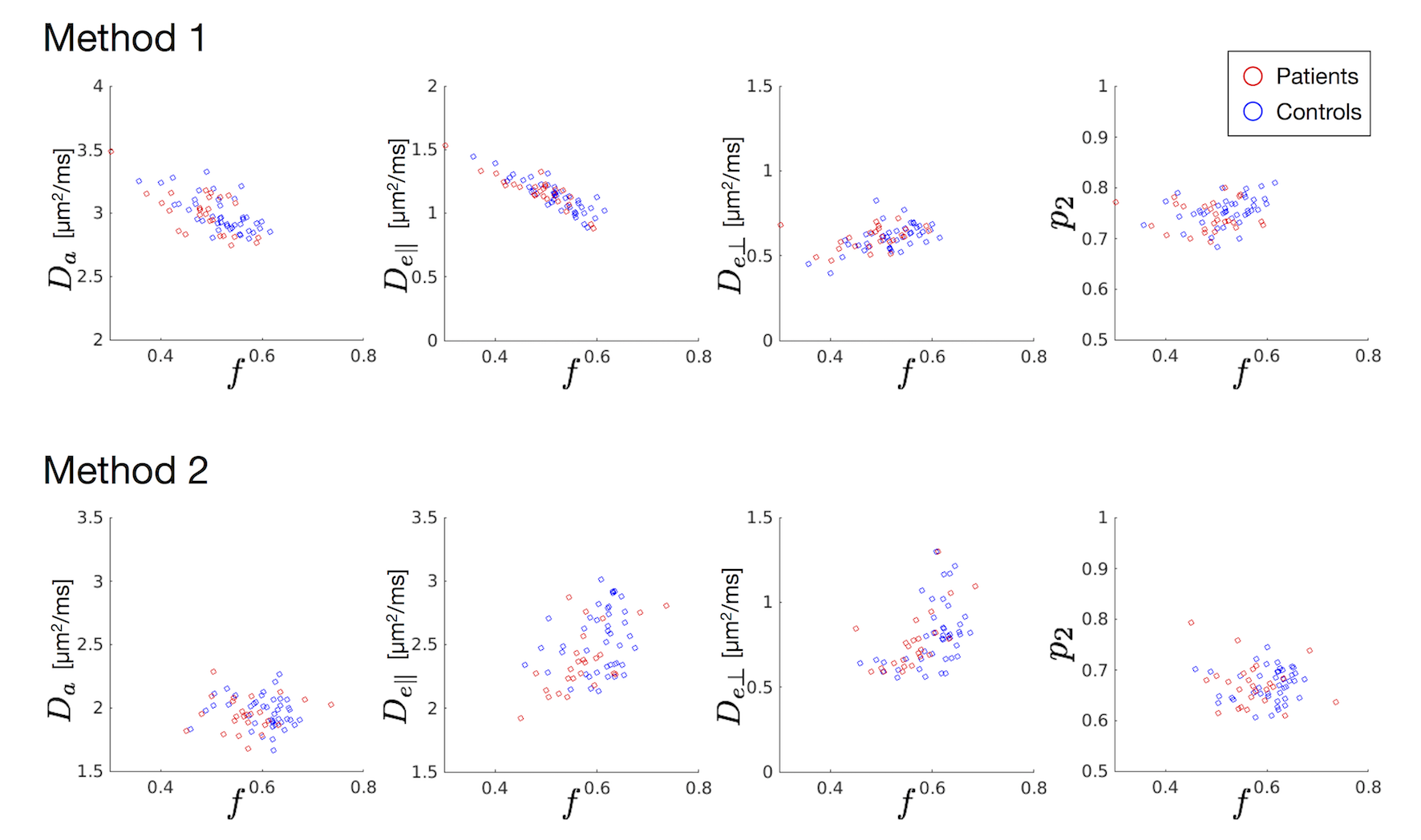


**Fig. S2** Correlations among the model parameters in Region II of the corpus callosum. The trend was similar in the other ROIs. The correlations were dependent on the methods used and closely resembled those induced by noise (Figure 4), suggesting these correlations are intrinsic to our parameter estimation and not much related to the biological reality.
